# Editorial: Exploring the physiological and molecular benefits of physical exercise in cardiovascular diseases

**DOI:** 10.3389/fphys.2026.1809357

**Published:** 2026-02-25

**Authors:** Alex Cleber Improta-Caria, Roque Aras Júnior

**Affiliations:** 1 Laboratory of Biochemistry and Molecular Biology of the Exercise, University of Sao Paulo (USP), São Paulo, Brazil; 2 Faculty of Medicine, Federal University of Bahia (UFBA), Salvador, Bahia, Brazil

**Keywords:** cardiovascular diseases, exercise training, molecular adaptations, physical exercise, physiological adaptations

Cardiovascular diseases (CVDs) are the leading cause of death worldwide (Stark et al., 2025). Several strategies are used to inhibit the development or progression of CVDs, and one of them is through exercise training (ExT). ExT is widely recognized as an excellent non-pharmacological tool for promoting various benefits to the cardiovascular (Improta-Caria et al., 2024), neurological (Roever et al., 2023), and other systems.

Many of the physiological benefits of ExT in individuals with CVDs have already been described in the literature (Hastings et al., 2024), however, the benefits at the molecular level still need to be further explored. Thus, in this special edition, we have gathered articles that address the impact of different types of ExT on the cardiovascular system and on CVDs, showing the effects of ExT at the molecular level.

ExT can be mainly divided into 2 different types: aerobic ExT (De Sousa and Improta-Caria, 2022) and resistance ExT (Paluch et al., 2024), and more recently, more evidence has emerged about high intensity interval training (HIIT) (Wewege et al., 2018). Aerobic ExT is the most suitable for individuals with CVDs. In this context, Improta-Caria et al. demonstrated that 5 weeks of aerobic ExT was able to reduce collagen content in the hearts of mice with chronic Chagas cardiomyopathy, as well as decrease collagen content in skeletal muscles. This beneficial effect of ExT in reducing collagen in cardiac tissue promoted improvement in cardiac arrhythmias, attenuating cardiac dysfunction in these animals. It is important to emphasize that chronic Chagas cardiomyopathy is a disease that induces an increased inflammatory and fibrotic profile in both cardiac and skeletal muscle (Meira et al., 2019), in addition to promoting severe arrhythmias and cardiac dysfunction (Bocchi et al., 2017).

In this context, ExT is a key tool in cardiac rehabilitation, both for chronic Chagas cardiomyopathy and other types of cardiomyopathies (Price et al., 2016). The work of You et al. demonstrated the importance of cardiac rehabilitation by bringing a new perspective based on integrative physiology. This interesting review demonstrated how ExT protects the heart, showing that the effects of ExT promote beneficial crosstalk between the heart and other organs such as skeletal muscle, brain, kidney, gut, liver, and adipose tissue, in addition to explaining some modifications in epigenetic regulation induced by ExT.

The modifications in epigenetic regulation induced by ExT are a new and increasingly evolving topic in current science. In this regard, Yuan et al. explained the impacts of ExT on the expression of microRNAs (miRNAs), and how these impacts on miRNA modulation promote cardioprotection, inducing beneficial changes in some important biological processes such as inflammation, oxidative stress, endothelial function, and cardiac remodeling. In addition to changes in miRNA expression, this important review explains that ExT also induces modifications in the expression of long non-coding chromosomes, DNA methylation, and histone acetylation, promoting improved mitochondrial function, reduced inflammation, decreased fibrotic profile, and improved cardiovascular resilience.

In this context, Silva et al. also explained the importance of ExT in inducing epigenetic changes, however, the authors focused on the effects of resistance training. This elegant review initially explains how resistance training promotes changes at the physiological level, promoting beneficial concentric cardiac hypertrophy, improved vascular function, reduced blood pressure, normalization of blood glucose levels, among others. Subsequently, the text discusses how resistance training induces epigenetic modifications, altering miRNA expression, promoting histone acetylation and DNA methylation, generating various changes in signaling pathways in both healthy individuals and individuals with cardiovascular diseases.

In addition to the benefits promoted by aerobic exercise and resistance training, this Research Topic also addressed the effects of high-intensity interval training (HIIT). Li and Dong showed that HIIT induces benefits in cardiovascular parameters by reducing systolic and diastolic blood pressure, modifying pulse wave velocity, and decreasing heart rate, improving vascular elasticity and endothelial function. In this sense, the interesting systematic review with meta-analysis by Zheng et al. shows that low-volume HIIT is a good and time-efficient strategy to efficiently improve the cardiovascular health of children and adolescents, significantly reducing body mass index, fat mass, waistline, weight, and improving VO2 max in these individuals.

Overall, the evidence gathered in this Research Topic represents valuable work that enriches the scientific literature on physiological and molecular adaptations induced by ExT. Further clinical and preclinical studies are essential for a deeper understanding of the various benefits orchestrated by different types of ExT on cardiovascular health and overall health, preventing the development and progression of CVDs.
